# Diagnosis and genotyping of African swine fever viruses from 2015 outbreaks in Zambia

**DOI:** 10.4102/ojvr.v83i1.1095

**Published:** 2016-04-29

**Authors:** Jonas Thoromo, Edgar Simulundu, Herman M. Chambaro, Liywalii Mataa, Caesar H. Lubaba, Girja S. Pandey, Ayato Takada, Gerald Misinzo, Aaron S. Mweene

**Affiliations:** 1 Department of Veterinary Microbiology and Parasitology, Sokoine University of Agriculture, Tanzania; 2 Department of Disease Control, The University of Zambia, Zambia; 3 Ministry of Fisheries and Livestock, Lusaka, Zambia; 4 Division of Global Epidemiology, Hokkaido University Research Center for Zoonosis Control, Japan

## Abstract

In early 2015, a highly fatal haemorrhagic disease of domestic pigs resembling African swine fever (ASF) occurred in North Western, Copperbelt, and Lusaka provinces of Zambia. Molecular diagnosis by polymerase chain reaction targeting specific amplification of p72 (*B646L*) gene of ASF virus (ASFV) was conducted. Fourteen out of 16 domestic pigs from the affected provinces were found to be positive for ASFV. Phylogenetic analyses based on part of the p72 and the complete p54 (*E183L*) genes revealed that all the ASFVs detected belonged to genotypes I and Id, respectively. Additionally, epidemiological data suggest that the same ASFV spread from Lusaka to other provinces possibly through uncontrolled and/or illegal pig movements. Although the origin of the ASFV that caused outbreaks in domestic pigs in Zambia could not be ascertained, it appears likely that the virus may have emerged from within the country or region, probably from a sylvatic cycle. It is recommended that surveillance of ASF, strict biosecurity, and quarantine measures be imposed in order to prevent further spread and emergence of new ASF outbreaks in Zambia.

## Introduction

African swine fever (ASF) is a highly contagious and fatal haemorrhagic disease of domestic pigs caused by a complex DNA virus of the genus *Asfivirus,* family *Asfarviridae*. Most isolates of ASF virus (ASFV) cause an acute haemorrhagic fever with mortality rates that can reach 100% in susceptible pigs. ASFV circulates in an ancient sylvatic cycle involving soft ticks of the *Ornithodoros* species and warthogs (*Phacochoerus aethiopicus*) as well as in domestic pig populations with or without involvement of *Ornithodoros* ticks; other wild suids, in particular bushpigs (*Potamochoerus larvatus, Potamochoerus porcus*) can act as asymptomatic reservoirs of the virus but are not associated with ticks and their role, if any, in the epidemiology of the disease is unknown (Muhangi 2014).

Although ASF is known to be endemic in sub-Saharan Africa (Penrith *et al*. 2013), it is occasionally introduced into new regions or continents. For example, in June 2007, ASF was introduced into the Republic of Georgia and subsequently spread into neighbouring countries including Armenia, Azerbajan, the Russian Federation, Ukraine, and Belarus (Beltrán-Alcrudo *et al*. 2008; Chapman *et al*. 2011). Recently, ASF has spread into some of the member states of the European Union including Poland, Latvia, Lithuania, and Estonia (Pejsak *et al*. 2014). These events stress the need for concerted international efforts in the control of ASF.

In Zambia, ASF was first reported in 1912 in the vicinity of Fort Jameson, now called Chipata, in Eastern Province (Wilkinson *et al*. 1988). Subsequently, there have been repeated reports of its occurrence in the same area, where it is now considered to be endemic. Laboratory confirmation was done on only five occasions between 1974 and 1983 (Wilkinson *et al*. 1988). However, a recent study confirmed an outbreak of ASF in Lusaka in October 2013 (Yabe *et al.* 2015). Although the Zambian government has endeavoured to control the disease, outbreaks continue to be reported across the country in non-endemic regions (Yabe *et al*. 2015). In early 2015, outbreaks of a haemorrhagic disease resembling ASF that killed a number of domestic pigs were reported in North Western, Copperbelt, and Lusaka provinces of Zambia. The present study was aimed at confirming and conducting genotypic characterisation of ASFVs involved in these outbreaks.

## Materials and methods

### Study area, sample collection, and processing

**FIGURE 1:**
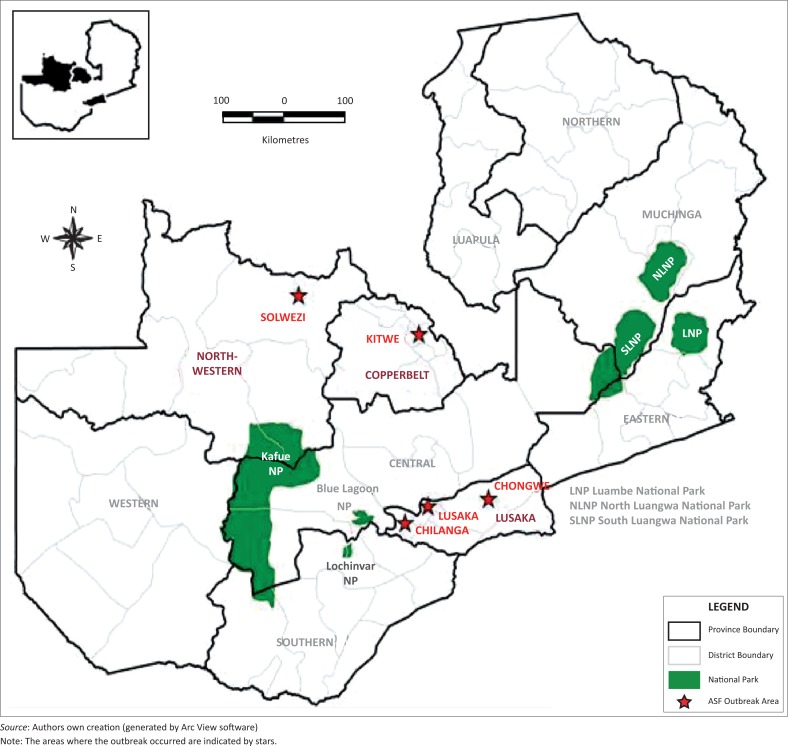
Map showing sampling locations for African swine fever in domestic pigs.

Tissue samples were collected from domestic pigs in districts with suspected ASF cases based on clinical signs, post-mortem lesions, and reports from local veterinary officials and farmers during January to April 2015. Samples were obtained from Kitwe district in the Copperbelt province; Chongwe, Chilanga (Linda area), and Lusaka (Chamba valley area) in Lusaka province; and Solwezi district in North Western province (Figure 1). Eight samples were collected from domestic pigs in Lusaka province, four samples came from North Western, and four from the Copperbelt provinces, making a total of 16. Tissues collected included mesenteric lymph nodes, spleen, kidney, and liver. Samples were collected in sterile tubes and transported on ice to the laboratory where they were stored at -80 °C until use. Tissues were homogenised in phosphate-buffered saline at a ratio of 1:10 (w/v). Tissue homogenates from each animal were pooled and used for laboratory diagnosis and molecular analyses.

### DNA extraction

One µL of Proteinase K (20 mg/mL) was added to 100 µL of 10% tissue homogenate followed by overnight incubation of the tissue-enzyme mixture at 55 °C. Then the tissue-enzyme mixture was heated at 95 °C for 10 min followed by centrifugation at 6000 g for 5 min. The supernatant containing DNA was collected and the pellet discarded.

### Diagnosis of African swine fever virus using polymerase chain reaction

ASFV DNA was detected by polymerase chain reaction (PCR) using PPA1/2 primers, as previously reported by Agüero *et al*. (2003). PCR was carried out in Gene Amp PCR System (Applied Bio-systems, Foster City, CA USA) using *Taq* polymerase (Thermoscientific, Waltham, MA USA), according to the manufacturer’s instructions. PCR products were separated by electrophoresis on a 1.5% agarose gel stained with GelRed nucleic acid stain (Phenix Research Products, Candler, USA).

### Genotyping of African swine fever virus

Genotyping of ASFV was performed by partial amplification of the p72 (*B646L*) and complete p54 (*E183L*) gene amplification using PCR. Primers including P72U/D and PPA89/722 were used in the amplification of p72 and p54 genes, respectively (Bastos *et al*. 2003; Nix *et al*. 2006). The amplification conditions previously described by Gallardo *et al*. (2009) were used in the present study. PCR products were purified from agarose gels using a NucleoSpin gel and PCR clean-up kit (Macherey-Nagel, Düren, Germany) and subjected to dideoxynucleotide cycle sequencing using Big Dye Terminator Cycle Sequencing Kit Version 3.1 (Applied Biosystems, Foster City, CA USA). Products from dideoxynucleotide cycle sequencing reaction were purified by ethanol precipitation and separated on a 3500 Genetic Analyzer (Applied Biosystems, Foster City, CA USA). Chromatograms for both the forward and the reverse primer reactions were read using Sequence Scanner v1.0 software (Applied Biosystems, Foster City, CA USA). The obtained sequences were deposited in GenBank/EMBL/DDBJ databases under accession numbers LC088171-LC088176.

### Data analysis

ASFV nucleotide sequences representing the 22 p72 genotypes (Boshoff *et al*. 2007) were obtained from GenBank database and analysed in MEGA 6.06 (Tamura *et al*. 2013) along with those determined in the present study. The p72 and p54 nucleotide sequences were aligned in MEGA 6.06 using Clustal W. Phylogenetic reconstruction was conducted using the neighbour-joining method. Phylogenetic tree reliability was supported using the bootstrap method with 1000 replications.

## Results

### Outbreak history

In Solwezi district in North western province, an outbreak of a pig disease similar to ASF was observed in January–February 2015 at a farm with a total of 66 pigs. Twenty pigs died as a result of the disease whereas 46 were depopulated by government. At another farm in the same district 7 pigs died from a similar disease and the rest (23 pigs) were depopulated by government. Epidemiological investigations revealed that all the pigs at the primary farm were brought from the Copperbelt province. During this time period, 4 pigs died from a herd of 68 at a farm in Kitwe district of the Copperbelt. The rest of the pigs (64) were depopulated by government officials. The origin or source of the disease was not established. In Chongwe district (Lusaka province), the disease came about after a boar was bought from Kaunda square in Lusaka from a farmer whose pigs had started dying and was selling them off. The farmer in question had initially bought the pigs from Chilanga district. This was during a time when it was not mandatory to test animals meant for breeding for ASF. The case was reported in April 2015. During the sampling period, more than 600 pigs died in Lusaka province including Chilanga (Linda area), Chongwe, and Lusaka (Chamba valley area) either through depopulation or from disease.

### Clinical signs and post-mortem findings

Clinical signs and gross pathological findings of the screened animals in this study were similar to those of ASF, and hence this disease was highly suspected. The animals presented with incoordination, inappetence, cyanosis of the ears, petechial haemorrhages on leg extremities, tachypnoea, and recumbence. On post-mortem examination pigs had oedematous lungs, fluids in body cavities, and haemorrhages and congestion in internal organs such as the spleen, lymph nodes, intestine, and kidneys.

### Detection of African swine fever virus genome

In total, 16 clinical samples from four different districts in Zambia were analysed using PCR. Out of the 16 samples, ASFV was detected in 14 samples using three different pairs of primers that specifically amplify different parts of ASFV genome including *B646L* and *E183L.*

### Nucleotide sequence and phylogenetic analysis

The p72 and p54 nucleotide sequences of ASFV detected in domestic pigs in Kitwe, Solwezi, Chongwe, Lusaka, and Chilanga were found to be 100% similar to each other.

**FIGURE 2:**
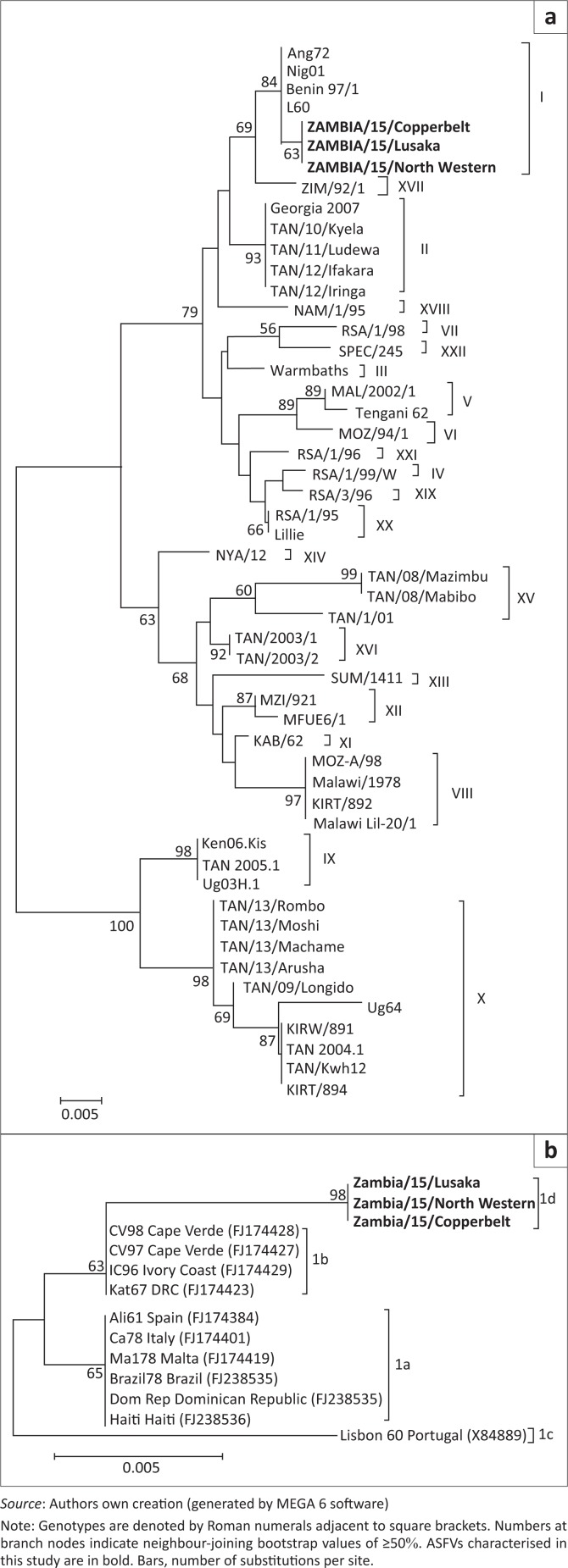
Phylogenetic relationships based on nucleotide sequences of part of p72 (a) and p54 (b) genes of African swine fever virus detected in tissues collected from diseased pigs in Zambia.

Phylogenetic analysis of the p72 gene sequences from this study clustered into genotype I (Figure 2a). Similarly, phylogenetic analysis of the *E183L* (p54) nucleotide sequences showed that the 2015 Zambian ASFV belonged to genotype Id (Figure 2b). Moreover, they were different from other genotype I ASFVs such as those from West Africa (genotype Ib) and America and Europe (genotype Ia and Ic) (Figure 2b).

## Discussion

Although Zambia has experienced many outbreaks of ASF, genetic characterisation of ASFVs involved in outbreaks has been conducted only for some viruses. Available genotyping studies were performed before the year 2008 (Lubisi *et al*. 2005, 2007; Nix *et al*. 2006). However, ASF outbreaks in domestic pigs have continued to occur in Zambia, including the one reported in 2013 (Yabe *et al*. 2015) and those reported in this study.

In this study, clinical and post-mortem findings consistent with those of ASF were observed suggesting that outbreaks of a highly fatal haemorrhagic disease afflicting domestic pigs in North Western, Copperbelt, and Lusaka provinces of Zambia could be ASF. ASFV infection of diseased pigs was confirmed through detection of different parts of the ASFV genome by PCR. The finding that nucleotide sequences of the p72 and p54 genes of ASFV under study were 100% identical suggests that these outbreaks were caused by the same virus. Phylogenetic analyses based on the p72 nucleotide sequences confirmed that the 2015 ASFV from Zambia belonged to genotype I (Figure 2a). Genotype I ASFVs have a wide geographical distribution and have been previously reported in Europe, South America, the Caribbean Islands, West Africa, East Africa, and southern Africa (Lubisi *et al*. 2005). Based on the finding that the p54 nucleotide sequences of 2015 Zambian ASFV belonged to the southern African genotype 1d, it seems likely that this virus may have originated from within the country (or possibly from the southern African region) and may not have been imported from other geographical locations.

Epidemiological data gathered from farmers and veterinarians among the different provinces of Zambia during the 2015 ASF outbreaks suggest that ASF spread within Zambia was facilitated by movement of infected pigs and/or pig products from affected areas to disease free areas. For instance, in Chongwe district (Lusaka province), the disease came about after a boar was bought from Kaunda square (Lusaka district) from a farmer who was selling off pigs because his pigs had started dying. Similarly, ASF broke out in Solwezi after a farmer purchased pigs from the Copperbelt province. It is therefore recommended that future control and prevention strategies of ASF in Zambia should focus on early detection through screening of the animals and timely confirmation of the disease, strict quarantine measures, biosecurity, stock movement restrictions including culling and proper disposal of infected and in-contact animals, and decontamination of affected premises.
